# Phonotactic Diversity Predicts the Time Depth of the World’s Language Families

**DOI:** 10.1371/journal.pone.0063238

**Published:** 2013-05-17

**Authors:** Taraka Rama

**Affiliations:** Språkbanken, Department of Swedish Language, Göteborg, Sweden; University of Warwick, United Kingdom

## Abstract

The ASJP (Automated Similarity Judgment Program) described an automated, lexical similarity-based method for dating the world’s language groups using 52 archaeological, epigraphic and historical calibration date points. The present paper describes a new automated dating method, based on phonotactic diversity. Unlike ASJP, our method does not require any information on the internal classification of a language group. Also, the method can use all the available word lists for a language and its dialects eschewing the debate on ‘language’ vs. ‘dialect’. We further combine these dates and provide a new baseline which, to our knowledge, is the best one. We make a systematic comparison of our method, ASJP’s dating procedure, and combined dates. We predict time depths for world’s language families and sub-families using this new baseline. Finally, we explain our results in the model of language change given by Nettle.

## Introduction

Glottochronology, as introduced by Swadesh [1], [2], is a method for estimating the split/divergence time of two phylogenetically related languages from their common ancestor. It makes use of Swadesh lists, which are short lists, usually 100–215 items of core vocabulary, supposed to be resistant to borrowing and is universal and culture-free.

Core vocabulary is supposedly more resistant to lexical replacement than other vocabulary items. There is an assumption of a universal constant rate of lexical change over time. The time depth of the point of split between two languages is proportional to the logarithm of lexical similarity. The lexical similarity between two languages is measured as the percentage of cognates, 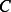, shared between the pair of languages. The time depth is estimated in units of 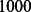 years using the following formula.
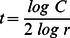
(1)


The constant 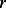 is experimentally determined by Lees [3] using 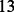 control cases.

Glottochronology was heavily criticized for several reasons, especially the following ones:

- The composition of the core vocabulary list is not objective. Only recently, in [4], [5] was the assumption of stability of the core vocabulary tested quantitatively for the worldwide language families.
- The rate of lexical replacement is not constant across different families or within the families. As demonstrated in [6], Icelandic has a relatively lower rate of lexical change and East Greenlandic Eskimo has a higher rate of lexical change than assumed by Lees [3].

The related work in the field of computational historical linguistics is described in the next subsection.

### Related Work

The last decade has seen a surge in the number of papers published in historical linguistics applying computational and statistical methods. This literature can be broadly classified into two areas.

One area of work, represented in [7], [4], [8], [9], [10], and [11] focuses on collecting word lists for various language families for attacking classical historical linguistics problems such as dating, internal language classification, and lexical stability.

The other area of work, represented by papers such as [12], [13], [14], [15], [16], and [17] is characterized by the application of quantitative methods to seek answers to questions also involving socio-historical processes, including the relations between language diversity, human population sizes, agricultural patterns and geographical origins of languages. It should be noted that this classification is not strictly mutually exclusive (See [18] for a survey of the computational, statistical and inter-disciplinary work on language dynamics and change). Of the several works cited above, those of [7], [19], [9] are relevant to this paper.

Grey and Atkinson [11] date the Indo-European family as 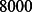 years old using a penalized maximum likelihood model which supports the Anatolian hypothesis of language spread. They use a binarily encoded character matrix (presence/absence of a cognate for a language; judged by comparative method) for Indo-European from Dyen et al. [20] for inferring the phylogenetic tree and dating its nodes.

A completely different approach is taken by the ASJP consortium for the automated dating of the world’s language families. ASJP (http://email.eva.mpg.de/wichmann/ASJPHomePage.htm) is a group of scholars who have embarked on an ambitious program of achieving an automated classification of world’s languages based on lexical similarity. As a means towards this end the group has embarked upon collecting Swadesh lists for all of the world’s languages. The database is described in the subsection ASJP Database below.

Holman et al. [9] collected calibration points for 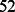 language groups from archaeological, historical and epigraphic sources. The intra-language group lexical similarity was computed using a version of the Levenshtein distance (LD). Levenshtein distance is defined as the minimum number of substitution, deletion and insertion operations required to convert a word to another word. This number is normalized by the maximum of the length of the two words to yield LDN, and finally the distance measure used, LDND (LDN Double normalized), is obtained by dividing the average LDN for all the word pairs involving the same meaning by the average LDN for all the word pairs involving different meanings. The second normalization is done to compensate for chance lexical similarity due to similar phoneme inventories between unrelated languages. Now, we describe the computation of average lexical similarity for a intra-language group using the Scandinavian calibration point. The Scandinavian language group has two sub-groups: East Scandinavian with 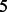 word lists and West Scandinavian with 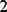 word lists. The internal classification information is obtained from *Ethnologue* [21]. The ASJP procedure sums the LDND of the 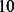 language pairs and divides them by 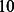 to yield an average LDND for Scandinavian language group. Then, they fit a ordinary least-squares regression model of the average lexical similarity as a predictor with time depth as the response variable. The regression yields a highly robust correlation of 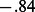. Finally, they use the fitted regression model to predict a language group’s ancestral time depth for different language families across the world.

Serva and Petroni [19] were the first to use LD to estimate the time-depth of a language family. But their experiments were focused on dating the root of the Indo-European tree. They primarily use Dyen et al.’s [20] IE database – augmented by some of their own data – for their experiments.

## Materials and Methods

### ASJP Database

The ASJP database ([22]; Expanded versions of the ASJP database are continuously being made available at http://email.eva.mpg.de/ *

wichmann/EarlierWorldTree.htm*) has 4817 word lists from around half of the languages of the world including creoles, dialects, artificial languages and extinct languages. We work with the version 13 database for comparability with the results given by the ASJP dating procedure. A language and its dialects is identified through a unique ISO 639-3 code given in *Ethnologue* [21]. The database also contains the languages’ genetic classification as given in WALS [23] and *Ethnologue* [21]. The database has a shorter version – the 40 most stable meanings empirically determined by [4] – of the original Swadesh list. A word list for a language is normally not entered into the database if it has less than 70% of the 40 items. For our experiments, we use a subset of the data obtained by removing all the languages extinct before 1700 CE.

The word lists in ASJP database are transcribed in ASJPcode [24]. ASJPcode consists of characters found on a QWERTY keyboard. ASJPcode has 34 consonant symbols and 7 vowel symbols. The different symbols combine to form complex phonological segments. Vowel nasalization and glottalization are indicated by * and ″, respectively. Modifiers ∼ and $ indicate that the preceding two or three segments are to be treated as a single symbol.

### ASJP Calibration Procedure

The motivation for and the details of the ASJP calibration procedure is outlined in this section. There are at least three processes by which the lexical similarity between genetically related languages decreases over time. Shared inherited words (cognates) undergo regular sound changes to yield phonologically less similar words over time (e.g. English/Armenian *two* ∼ *erku* ‘two’; English/Hindi *wheel* ∼ *chakra* ‘wheel’). Words can also undergo semantic shift or are replaced through copying from other languages causing a decrement in the lexical similarity between related languages. LDND is designed specifically to capture the net lexical similarity between languages related through descent.

The ASJP’s date calibration formula is similar to that of glottochronology (1). Eqn. 1 implies that the ancestral language is lexically homogeneous at 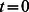. This formula is modified to accommodate lexical heterogeneity of the ancestral language at time zero by introducing 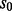, representing average lexical similarity at 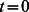 of the language groups’ ancestral language. The cognate proportion 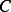 is replaced by the ASJP lexical similarity defined as 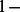
LDND. The formula then looks as in (2):
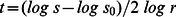
(2)


The values of 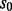 and 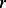 are empirically determined by fitting a linear regression model between the 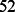 language groups’ time depth (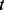) and their lexical similarity (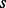). The intra-language group similarity is defined as the average pairwise lexical similarity between the languages belonging to the coordinate subgroups at the highest level of classification. Eqn. 2 and the negative correlation implies that log lexical similarity has an inverse linear relationship with time depth.

The next subsection describes our findings on the relation between language group diversity and the age of the group.

### Language Group Size and Dates

As mentioned earlier, the ASJP consortium [9] collected common ancestor divergence dates for 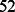 language groups, based on archaeological, historical or epigraphic evidence. Written records can be used to determine the date of divergence of the common ancestral language reliably. The recorded history of the speakers of the languages can be used to determine the divergence dates based on major historical events. Since written records do not exist for temporally deep language families, the date for the common ancestor must often be inferred from archaeological sources.

Archaeological dates can be determined on the basis of traceability of the proto-language’s reconstructed words to excavated material objects. Dates can also be inferred if loanwords can be correlated with historical or archaeological events. The process of compiling calibration points was extremely careful and archaeological calibration points were only included if they were non-controversial. Specifically, any purely glottochronologically determined date was excluded from the sample.

A description of the sources of the dating points, the language groups’ subgrouping adopted for computing the ASJP similarity, and also the ASJP similarity is available in the original paper. We wrote a python program to automatically extract the languages for a given group based on the description given in the original paper. The data for number of languages, calibration date, type of the date, the genetic family, the mode of subsistence (pastoral or agriculture; from the compilation of [16]), and the geographic area (based on the continents Eurasia, Africa, Oceania, the Americas) for each language group are given in Table S1 in File S1.

**Figure 1:**
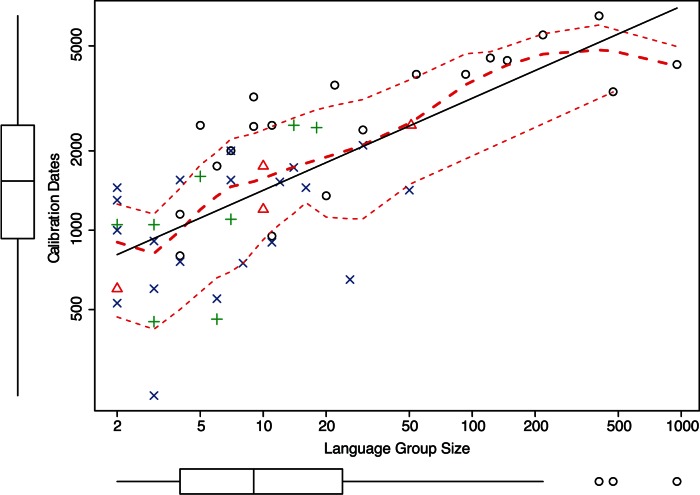
Calibration dates against the number of languages in a language group. 
s are archaeological, 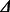
s are archaeological and historical, 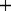
s are epigraphic and 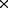
s are historical dates.

First, we tested whether the sheer size of the language group (LGS) is related to the calibration dates. The size was determined by counting the number of languages in each language group, using *Ethnologue* [21]. A scatter plot with time depth against LGS (on a log-log scale) shows a linear relationship. The regression, shown in Fig. 1, is 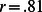 and highly significant 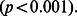 The linear relationship is shown by a solid straight regression line. The younger dates are closer to the regression line than the older archaeological dates. Fig. 1 also displays the box plots of each variable along its axis. The box plot of LGS shows three outliers for groups larger than 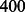, which are farther away from the rest of the dates but not from the regression line. The dotted line is the locally fitted polynomial regression line (LOESS; with degree 2). The LOESS line stays close to the linear regression line confirming that using a linear regression analysis is appropriate. The square root of the variance of the residuals for the LOESS line is also shown as dotted lines on both the sides of the LOESS line.

Although this approach does not require the subgrouping information it is not without problems. The ASJP database often has word lists from dialects of a single language. The ASJP calibration procedure described in ASJP calibration procedure subsection above includes all the dialect word lists for a single language identified by its ISO code. Similarly, the LGS variable also counts the total number of available word lists for a language group as its size (We obtain a Pearson’s 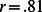 when LGS variable is counted as the number of languages given in *Ethnologue* [21]). Nettle [14] summarizes the ‘language’ vs. ‘dialect’ judgmental difficulties when adopting language counts from *Ethnologue* for quantifying language diversity (number of languages spoken per unit area). In another work, Nordhoff and Hammarström [25], use the term ‘doculet’ to indicate a linguistic variety identified in its descriptive resource. They use this definition to list various language variants in their database *Langdoc*.

In this paper, we follow a different approach which has the following advantages. It requires neither the internal classification information of a language group nor the judgment of language vs. dialect. The approach can use all the available word lists for a language and its dialects identified by a unique ISO 639-3 code. Our approach is described in the next subsection.

### Calibration Procedure

In this section, we describe the computation of N-gram diversity and the model selection procedure. The model is run through a battery of tests to check for its robustness. We mix the N-gram model with the ASJP dates to produce a better baseline. Finally, we use the N-gram model to predict the dates for world-wide language groups as given in *Ethnologue*.

### N-grams and Phonotactic Diversity


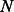
-grams are ubiquitous in natural language processing (NLP) and computational linguistics, where they are used in systems ranging from statistical machine translation to speech recognition, but they are relatively unknown in historical linguistics. 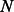-grams are defined as a subsequence of length 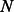 from a sequence of items. The items could be part-of-speech tags, words, morphemes, characters or phonemes. 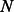-grams were originally introduced as a probabilistic model for predicting the next linguistic item, given a history of linguistic items [26]. The word “oxen” has four letter 1-grams ‘o’, ‘x’, ‘e’, ‘n’; three letter 2-grams ‘ox’, ‘xe’, ‘en’; two letter 3-grams ‘oxe’, ‘xen’ and one letter 4-gram ‘oxen’. In general, any sequence of length 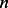 has 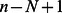 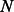-grams. The number of 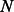-grams can similarly be calculated for a word in an ASJP word list for a given language. It has to be noted that the word initial and final 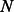-grams are not treated specially.

Having introduced 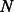-grams, we now define the phonological diversity of a language and show how it can be computed using 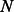-grams. Phonological diversity for a language is defined as the size of its phoneme inventory. In a similar fashion, the phonotactic diversity is defined as the total number of possible phoneme combinations in a language. For a language, the 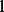-gram diversity (computed from a sufficiently long random list of phonetically transcribed words) is the same as phonological diversity. Extending it further, the phonotactic diversity can be computed as the 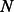-gram diversity 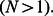 Given that the ASJP database (with its wide coverage) is a database of relatively short, 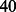-item word lists, it needs to be investigated whether the total number of unique phonological segments represented in the 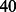 item word list can be used as a proxy for the actual phoneme inventory of a language.

Wichmann et al. [27] report a strong positive linear correlation of 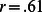 between the phoneme inventory sizes for a sample of 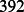 of the world’s languages, from the UPSID database [28] and the number of phonological segments (which is the same as the 1-gram diversity) represented in word lists for the corresponding languages in the ASJP database. The mean ratio of the ASJP segment size to the UPSID inventory size is 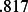 and the standard deviation is 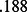. Also, there is a small correlation (Pearson’s 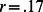) between the size of the word list, which can vary from 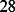 to 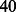, and the number of ASJP phonological segments. This puts us on a solid ground before proceeding to use 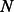-grams, extracted from the word lists, for purposes of calibrating dates.

The wide coverage of the ASJP database allows us to provide reasonable relative estimates of the total number of phonological sequences (using ASJPcode) present in the world’s languages. Since ASJP modifiers ∼ and 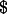 combine the preceding two or three symbols and there are **41** ASJP symbols in total, the number of theoretically possible phonological sequences is: 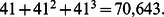 But the total number of ASJP sequences varies from 500 to 600 across all languages in the database depending on the criterion for extracting languages from the ASJP database.

The 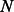-gram 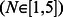 diversity of a language group is defined as the set of all the combined unique phonological segments of length 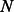 for the languages in the group. One might assume that 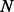-grams are not a signature of a language group or, in other words, that 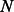-grams do not distinguish unrelated language families from each other. However, it can be empirically established that 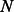-grams are more successful in distinguishing unrelated languages from each other than LDND. Wichmann et al. [7] devised a measure called *dist* (*Dist* of a family is defined as the difference between intra-family distance and inter-family distances divided by the standard deviation of the inter-family distances) for measuring the efficacy of a lexical similarity measure (in this case LDND vs. LDN) in distinguishing related languages vs. unrelated languages. In a separate experiment, which we will only briefly summarize here, using ASJP data from 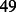 of the worlds’ language families, we employed a 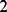-gram based measure, *Dice* (Between two strings: defined as twice the number of shared bigrams (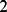-grams) divided by the total number of bigrams), for quantifying the distance between the language families and observed that it outperforms LDND in terms of *dist*. This empirical result shows that the set of 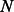-grams of a language family is a genetic marker for identifying related vs. unrelated languages. In the rest of the paper, 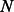-gram diversity implies the count of unique 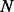-gram types present in a language group

## Results and Discussion

**Figure 2:**
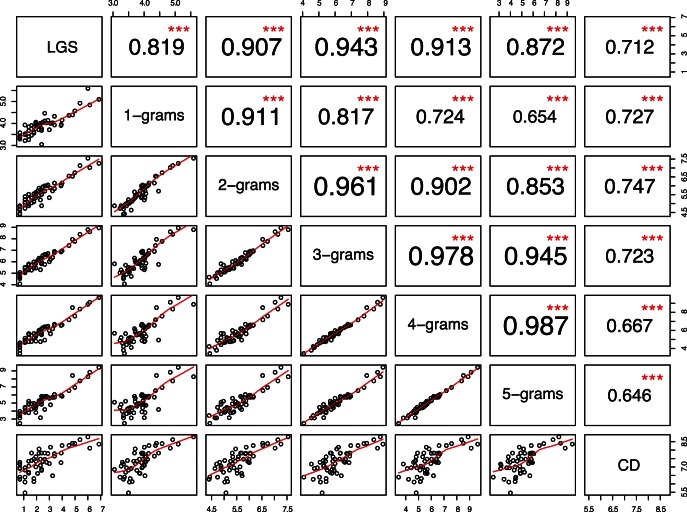
Pairwise scatterplot matrix of group size, *N*-gram diversity and date; the lower matrix panels show scatterplots and LOESS lines; the upper matrix panels show Spearman rank correlation (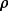) and level of statistical significance (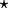). The diagonal panels display variable names. All the plots are on a log-log scale.

Objective judgment of shared inheritance of words in related languages becomes increasingly difficult due to the phonological distinctions accumulated over time. We hypothesize that 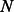-gram diversity for a language group is a non-decreasing function of time. To verify our hypothesis we check the nature of relationship between 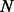-grams and dates. The last row in Fig. 2 shows the scatterplots of calibration dates (CD; given in Table S1 in File S1) vs. 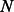-grams. The last column of the upper triangular matrix displays significant correlations and the highest correlation between 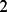-grams and CD. Both 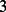-grams and 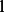-grams show a similar correlation with CD whereas, 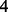-grams and 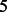-grams show a lower but a similar correlation. Another non-parametric test, Kendall’s 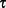, between the 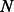-gram diversity and CD produces a relatively lower but highly significant correlation (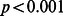). The highly significant 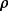 for different 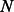-grams shows that the hypothesis holds for different orders of 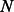-grams.

Further, there is a highly significant 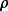 between 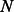-gram diversity and group size, as displayed in Fig. 2. There is a strong correlation between group size and 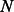-grams (greater than 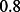 for all 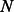). 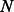-grams have a highly significant correlation (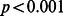) with each other. Deciding on the optimal value of 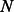 for the purpose of date calibration is a tricky issue. The LOESS lines for 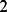
- and 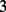-grams are nearly straight lines compared to the rest of 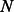-grams. There needs to be solid evidence for choosing 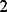
- and 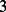-grams over the rest of 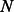-grams. We use the *AIC* measure (Akaike information criterion) coupled with further tests for selecting the appropriate value of 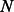. AIC is a relative measure of goodness for model selection. This measure is the negative sum of two components: the number of estimated parameters and the likelihood of the model. The number of parameters is the same across all the 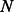-gram models. The lower the AIC, the better is the model. The AIC values for different 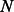-grams are given in Table 1. The values suggest that 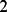-grams followed by 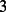-grams are the best fitting models. We employ a generalized linear model (Exponential family and log as link function; implementation available as *glm* function in R [29]) with Calibration Dates as the response variable and 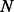-grams as predictors.

**Table 1:** The AIC score for each *N*-gram model is displayed in second column.

N	AIC
1	838.05
2	830.52
3	834.84
4	842.84
5	846.08

The significance scores for each model compared to the null model are based on a 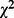 test (df = 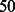). All the residual deviance scores are significant at a level of 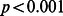.

Since all calibration dates greater than 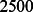 BP are archaeological, as an extra caution, ASJP tests the significance of the membership of a calibration date in one of the three groups (historical, epigraphic, archaeological) using a one-way analysis of variance (ANOVA). ANOVA tests whether the membership of a date in a group causes bias in the prediction by each 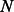-gram model. The calibration dates are grouped by type of dates, language family, geographical area and mode of subsistence. The data for these groups is available in Table S1 in File S1. Table 2 gives the results of the ANOVA analysis for various groups. The first column shows the group of the date. The second and third columns show the 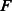-score for algebraic and absolute percent differences for all the 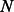-grams. The fourth column shows the degrees of freedom. The algebraic and absolute percent differences are computed as the percentage of algebraic and absolute residual values to the predicted values.

**Table 2:** 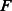
-score for algebraic and absolute percentage differences.

Group	F, algebraic					F, absolute					df
	1	2	3	4	5	1	2	3	4	5	
Type of date	7.38†	6.535†	3.217	3.014	3.206	0.455	1.268	2.357	1.766	1.423	3, 48
Language family	0.61	0.938	1.515	1.441	1.297	0.572	0.501	1.074	1.049	0.77	16, 35
Geographical area	1.148	1.019	0.533	0.518	0.368	0.093	0.018	0.677	0.603	0.431	3, 48
Mode of subsistence	2.553	4.152	4.887	2.91	1.988	0.390	0.272	1.164	0.173	0.04	1, 50

The significant scores are represented by a †.

Both algebraic and absolute percentages are tested against a significance level of 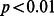. The test suggests that the predicted dates of 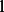-grams and 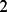-grams are biased in terms of type of the dates. The test suggests that the bias is with respect to archaeological class of dates. All the other values are non-significant and suggest that there is no difference across the groups. Thus, the ANOVA analysis suggests that the 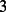-gram dates are more robust than 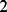-gram dates and are unbiased with respect to the groups.

We now test the validity of the assumptions of the regression analysis through the standard diagnostic plots, given in Section S2– figures S1, S2, S3, S4, and S5 in File S1. The diagnostic plots of the 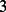-gram model in Fig. S3 in File S1 suggest that there has been no violation in the assumptions of regression analysis. The scatterplot between the predicted values and the residuals do not show any pattern. The residuals are normally distributed and the plot suggests that Dardic and Southwest Tungusic groups are the most deviating points. The normality assumption of the residuals is further tested through a Kolmogorov-Smirnov test (KST). KST tests against the null hypothesis that the residuals are distributed normally under a significance criterion of 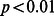. The test gives 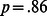 suggesting that we can retain the null hypothesis of normality. The ASJP dates for Dardic is underestimated by 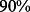 and overestimated for Southwest Tungusic by 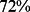. The 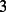-gram dates for Dardic and Southwest Tungusic are 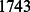 BP and 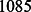 BP, respectively. It is not clear why there is such a huge discrepancy for these dates. The influential and leverage points are identified in subplot 3 (in Fig. S3 in File S1). The diagnostic plot does not suggest any influential points whereas there seems to be at least five high leverage points in the plot. The leverage points are identified as Benue-Congo, Eastern Malayo-Polynesian, Ga-Dangme, Indo-European and Malayo-Polynesian. All these points are archaeological and exceed a time depth of 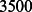 years (except for Ga-Dangme which is both archaeological and historical and only 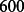 years old). As a matter of fact, the absolute percentage difference with respect to ASJP dates are as follows: –32,+12,–37,–26 and –41.

**Figure 3:**
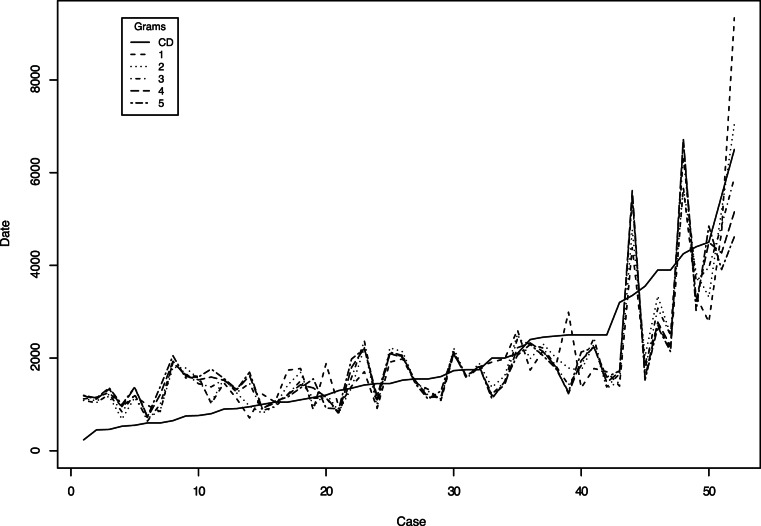
Comparing predicted dates for various n-grams.

Summarizing the regression analysis, there is a strong correlation of 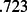 between the logarithm of 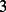-gram diversity and the calibration dates. We tested the assumptions of regression analysis and find that they are not violated. The 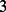-gram diversity reflects the net phonotactic diversity accumulated or lost in a language group over time. The predictions of all the 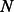-gram models and the respective calibration date are presented in Fig. 3.

The current findings can be explained in the terms of the basic model of language change described in [14]. In this model, languages diverge through imperfect replication of linguistic items amplified by selectional pressure and geographic isolation. Selectional pressures, namely social and functional selection, operate in the selection of the language variants generated through imperfect learning and the learner’s performance in this language. 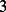-grams are a proxy for phonotactic diversity. The difference in phonotactic diversity between two languages represents the net result of phonological erosion, morphological expansion and fusion the language has undergone since its divergence from its most recent shared ancestor. The correlation between 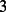-grams and time depth is just the reflection of this strong relation with net phonotactic diversity.

**Figure 4:**
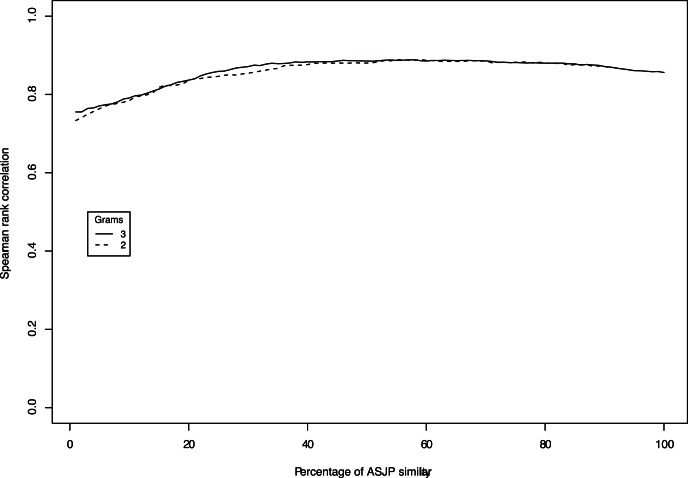
Combining ASJP with -grams and 3-grams: The ASJP dates are combined with 2-gram dates and 3-gram dates in different proportions ranging from 1% to 100% at an interval of 1.2

Since ASJP dates and 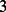-gram dates use different information from the same database, it would be interesting to see how the mixture of the predictions of the two models fare against the calibration dates. Each ASJP date is combined with a 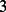-gram date using the following formula:
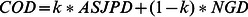
(3)where 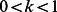, ASJPD is a ASJP date, NGD is either 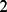-gram or 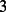-gram dates and, COD is a combined date. For a value of 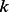, ranging from 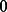 to 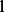, the value of 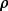 between COD and calibration dates is plotted in Fig. 4. The horizontal axis displays the scaled 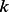 ranging from 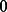 to 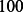. Fig. 4 shows that there is a modest, but steady increase in the correlation when ASJP dates are combined with 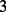-gram dates. The correlation increases until 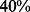 and then remains stable from 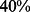 to 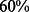. Both 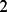-grams and 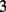-grams show the same trend. This indicates that a combination of the predictions indeed works better than individual models for the uncalibrated language families of the world. The optimal combination for 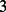-grams is obtained at 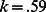.

The effect of mixing of 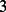-gram dates with ASJP dates is tested in Table 3. Table 3 gives a comparison of ASJP dates, 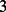-gram dates, and combined dates in terms of: sum and mean of absolute discrepancy, number of languages off by 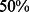 and 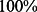, and 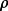. The ASJP analysis gave an upper bound of 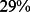 on the expected discrepancy between ASJP dates and the true dates for different language groups. We observe that the average of the absolute percentage discrepancy of combined dates (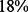) falls within the range of ASJP discrepancy. Clearly combined dates outperforms both the ASJP and 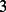-gram model’s methods. 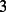-gram dates have the advantage that they neither requires subgrouping information nor the distinction between ‘language’ and ‘dialect’ but does not have the same 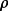 as ASJP dates. Combined dates performs the best but is the most complicated and has the disadvantages of ASJP dating.

**Table 3:** A comparison of different dating methods.

Measurement	ASJP	3-grams	combined
Sum of absolute discrepancy	1523	1815	927
Mean of absolute discrepancy	29	34	18
Off by 50%	5	13	2
Off by 100%	1	1	0
Spearman’s 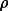	.86	.72	.89

### Worldwide Date Predictions

Finally, we predict time depths for the world’s language families, as given in *Ethnologue*, using the 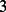-gram model. A combined date is given through Eq. 3. Both the predicted and the combined dates are given in Tables S2, S3, S4, S5, S6 (Section S3) in File S1. Each table presents the dates for all language families belonging to a geographical area – as defined in Materials and Methods section. The first column of each table shows the name of a language family and its subgroups (if any). For each language family, a subgroup and its further internal classifications are indented. For the sake of comparison, we give dates only for those families and subgroups given by ASJP [9]. The second column in each table shows the number of languages for a subgroup. The third and fourth columns show the ASJP dates and the 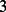-gram predicted dates. The fifth column shows the combined date, computed using Eq. 3. Whenever the ASJP date is missing for a language group we did not compute a combined date.

We now comment on the level of agreement found between ASJP dates and 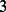-gram dates in Tables S2, S3, S4, S5, S6 in File S1 and try to explain the differences in terms of known linguistic or geographic factors. Except for Khoisan, the ASJP dates as well as 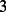-gram dates are quite similar. The language families Afro-Asiatic, Nilo-Saharan, and Niger-Congo are quite old and here the dates are similar. There is an enormous difference between the two dates for Khoisan. ASJP predicts 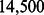 years as the time depth of Khoisan family whereas 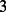-grams predict a shallower date (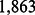 years). This huge disagreement could be attributed to the many-to-one mapping of click consonants by ASJP code. Additionally, ASJP [9] noted that some of the family classifications given in *Ethnologue* are controversial. Such a huge time gap could be a result of a lack of consensus in the general definition of a language family.

There is a relatively minor difference between the dates in [9] and 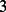-gram dates for the well-established language families of Eurasia such as Austro-Asiatic, Dravidian, Indo-European, Sino-Tibetan, and Uralic (Table S3 in File S1). Both models predict similar dates for Eurasian language families. The dates for languages of Pacific area is given in Table S4 in File S1. For Austronesian, a large language family (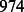 languages) in the Pacific area, the ASJP and 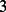-gram dates are 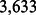 and 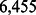 years, respectively. The combined date of Austronesian family is 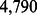 years which is fairly close to the age given by [30], 5,100 years.


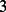
-gram dates and ASJP dates differ greatly for nearly all the language families of North America (Table S5 in File S1). For instance, ASJP [9] predict a time depth of 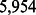 years for Algic whereas 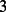-grams predict 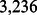 years. The 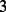-gram dates and ASJP dates differ by a few decades for the Mixe-Zoque and Mayan families, which are spoken in Middle America. A similar kind of difference is evident for a majority of South American languages (Table S6 in File S1). In summary, the ASJP and 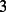-gram dates’ differences cannot be explained in terms of geographical areas. A huge gap between ASJP and 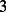-gram dates, such as Khoisan, might be a potential signal for a phantom phylogeny.

### Conclusion

In this paper we replicated the ASJP consortium’s process of extracting data representative of 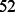 language groups for the use of calibrating linguistic chronologies. We proposed 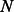-gram diversity as a measure of phonotactic diversity and found that 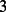-gram diversity had a significant correlation of 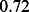 with calibration dates. The most important finding was that a combination of ASJP lexical similarity and 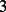-gram diversity, currently, is the best baseline for predicting the time depths for a language family. Finally, time depths for worldwide language families were predicted and combined with ASJP dates. The new dates are provided in Section S3 in File S1.

## Supporting Information

File S1 **Table S1, In column Type: A is archaeological, AH is archaeological and historical, H is historical and E is epigraphic calibration points.** In column Mode of subsistence: AGR is agricultural and PAS is foraging and pastoral. **Figure S1,** Diagnostic plots for 1-grams. **Figure S2,** Diagnostic plots for 2-grams. **Figure S3,** Diagnostic plots for 3-grams. **Figure S4,** Diagnostic plots for 4-grams. **Figure S5,** Diagnostic plots for 5-grams. **Table S2,** Dates for language groups of Africa. **Table S3,** Dates for language groups of Eurasia. **Table S4,** Dates for language groups of Pacific. **Table S5,** Dates for language groups of North and Middle America. **Table S6,** Dates for language groups of South America.
(PDF)
