# Supplementary material for: Phonotactic Diversity Predicts the Time Depth of the World’s Language Families
Source: PLoS One. 2013 May 17;8(5):e63238. doi: 10.1371/journal.pone.0063238 (PMC3656872; doi:10.1371/journal.pone.0063238)
Supplement: File S1 — Table S1, In column Type: A is archaeological, AH is archaeological and historical, H is historical and E is epigraphic calibration points. In column Mode of subsistence: AGR is agricultural and PAS is foraging and pastoral. Figure S1, Diagnostic plots for 1-grams. Figure S2, Diagnostic plots for 2-grams. Figure S3, Diagnostic plots for 3-grams. Figure S4, Diagnostic plots for 4-grams. Figure S5, Diagnostic plots for 5-grams. Table S2, Dates for language groups of Africa. Table S3, Dates for language groups of Eurasia. Table S4, Dates for language groups of Pacific. Table S5, Dates for language groups of North and Middle America. Table S6, Dates for language groups of South America. (PDF) [file pone.0063238.s001.pdf]

1 Supplementary Information

1.1 Data

Language group	Number of languages	Calibration date	Type	Family name	Mode of subsistence	Geographic area
Benue-Congo	404	6500	A	Niger-Congo	AGR	Africa
Brythonic	2	1450	H	Indo-European	AGR	Eurasia
Central SouthernAfrica Khoisan	7	2000	A	Khoisan	PAS	Africa
Cham	2	529	H	Austronesian	AGR	Oceania
Chamic	7	1550	H	Austronesian	AGR	Oceania
Chinese	7	2000	H	Sino-Tibetan	AGR	Eurasia
Cholan	5	1600	E	Mayan	AGR	Americas
Common Turkic	50	1419	H	Altaic	AGR	Eurasia
Czech-Slovak	2	1050	E	Indo-European	AGR	Eurasia
Dardic	22	3550	A	Indo-European	AGR	Eurasia
East Polynesian	11	950	A	Austronesian	AGR	Oceania
East Slavic	4	760	H	Indo-European	AGR	Eurasia
Eastern Malayo-Polynesian	472	3350	A	Austronesian	AGR	Oceania
English-Frisian	4	1550	H	Indo-European	AGR	Eurasia
Ethiopian Semitic	18	2450	E	Afro-Asiatic	AGR	Africa
Ga-Dangme	2	600	AH	Niger-Congo	AGR	Africa
Germanic	30	2100	H	Indo-European	AGR	Eurasia
Goidelic	3	1050	E	Indo-European	AGR	Eurasia
Hmong-Mien	14	2500	E	Hmong-Mein	AGR	Eurasia
Indo-Aryan	93	3900	A	Indo-European	AGR	Eurasia
Indo-European	218	5500	A	Indo-European	AGR	Eurasia
Indo-Iranian	147	4400	A	Indo-European	AGR	Eurasia
Inuit	4	800	A	Eskimo-Aleut	PAS	Americas
Iranian	54	3900	A	Indo-European	AGR	Eurasia
Italo-Western Romance	12	1524	H	Indo-European	AGR	Eurasia
Ket-Yugh	2	1300	H	Yeniseian	PAS	Eurasia
Maa	3	600	H	Nilo-Saharan	AGR	Africa
Malagasy	20	1350	A	Austronesian	AGR	Oceania
Malayo-Chamic	30	2400	A	Austronesian	AGR	Oceania
Malayo-Polynesian	954	4250	A	Austronesian	AGR	Oceania
Maltese-Maghreb Arabic	3	910	H	Afro-Asiatic	AGR	Africa
Mississippi Valley Siouan	9	2475	A	Siouan	PAS	Americas
Mongolic	8	750	H	Altaic	AGR	Eurasia
Northern Roglai Tsat	2	1000	H	Austronesian	AGR	Oceania
Ongamo-Maa	4	1150	A	Nilo-Saharan	AGR	Africa
Oromo	6	460	E	Afro-Asiatic	AGR	Africa
Pama-Nyungan	122	4500	A	Australian	PAS	Oceania
Romance	14	1729	H	Indo-European	AGR	Eurasia
Romani	26	650	H	Indo-European	AGR	Eurasia
Sami	6	1750	A	Uralic	PAS	Eurasia
Scandinavian	7	1100	E	Indo-European	AGR	Eurasia
Slavic	16	1450	H	Indo-European	AGR	Eurasia
Sorbian	3	450	E	Indo-European	AGR	Eurasia
Southern Nilotic	11	2500	A	Nilo-Saharan	AGR	Africa
Southern Songhai	6	550	H	Nilo-Saharan	AGR	Africa
Southwest Tungusic	3	236	H	Altaic	AGR	Eurasia
Swahili	10	1200	AH	Niger-Congo	AGR	Africa
Temotu	9	3200	A	Austronesian	AGR	Oceania
Tupi-Guarani	10	1750	AH	Tupi	AGR	Americas
Turkic	51	2500	AH	Altaic	AGR	Eurasia
Wakashan	5	2500	A	Wakashan	PAS	Americas
Western Turkic	11	900	H	Altaic	AGR	Eurasia

Table S 1. In column “Type”: ‘A’ is archaeological, ‘AH’ is archaeological and historical, ‘H’ is historical and ‘E’ is epigraphic calibration points. In column “Mode of subsistence”: ‘AGR’ is agricultural and ‘PAS’ is foraging and pastoral.

1.2 Diagnostic Plots

In this section, we present the four standard diagnostic plots for a linear regression analysis. Each plot has four sub-plots. The sub-plots from left-to-right in each row are summarized as followed:

- The scatter plot of the residuals vs the predicted value on a log scale.
- The residuals fitted against a standard normal distribution for testing the normality assumption of the residuals.
- A scatterplot showing the Cook's statistic vs. the leverage of each observation. Cook statistic suggests any points which influence the estimation of the regression parameters through a jackknifing procedure. The leverage points are those observations whose omitting influences the error value.
- A case plot of the Cook's statistic.

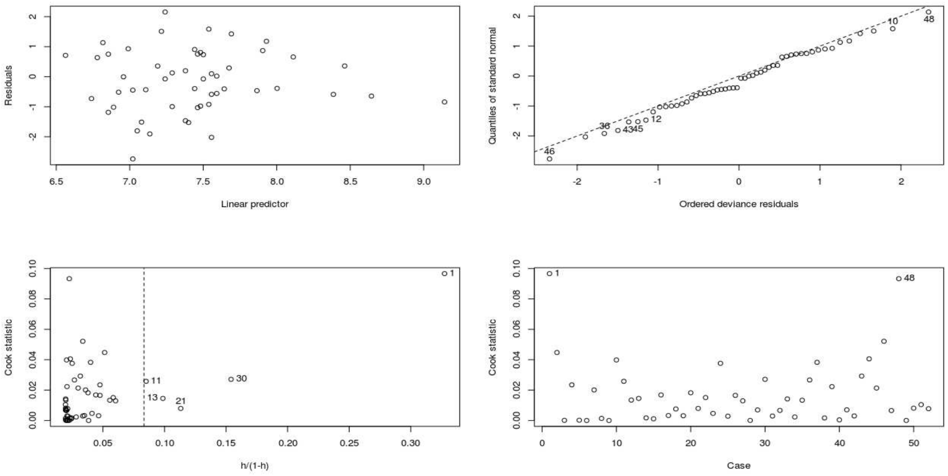

Figure S 1. Diagnostic plots for 1-grams

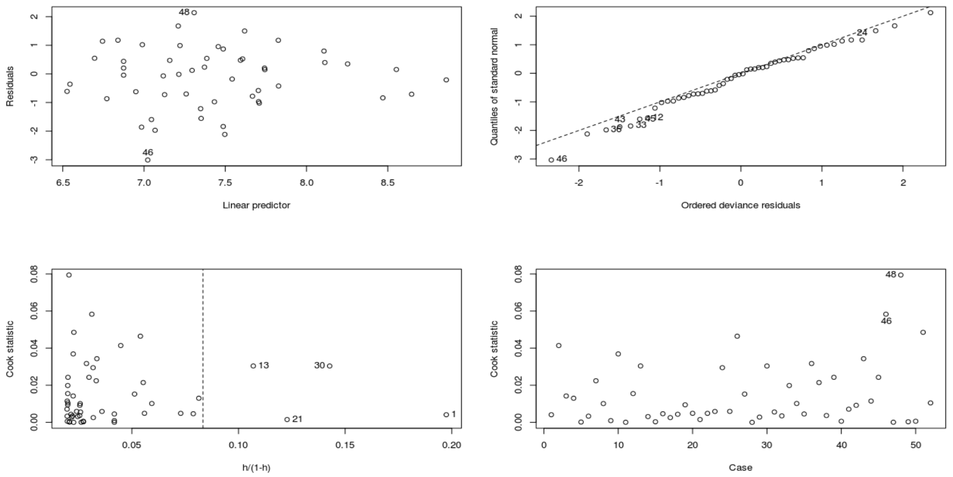

Figure S 2. Diagnostic plots for 2-grams

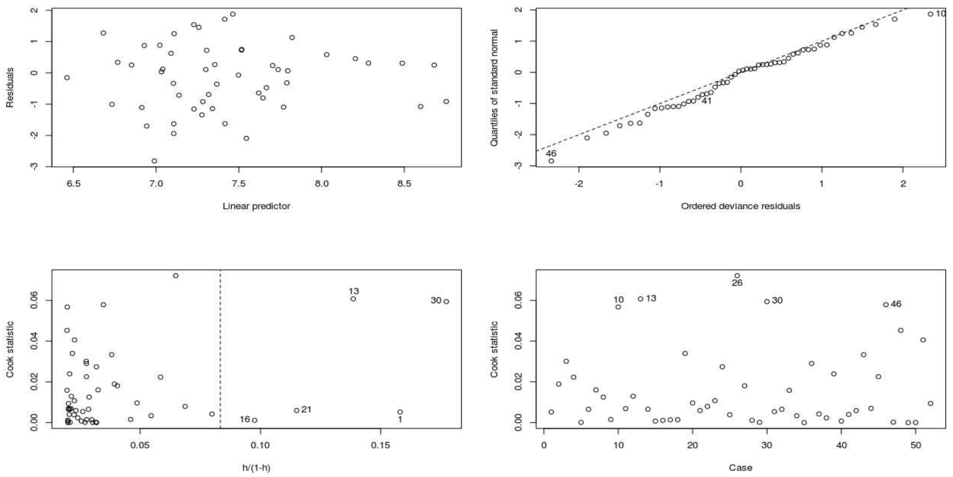

Figure S 3. Diagnostic plots for 3-grams

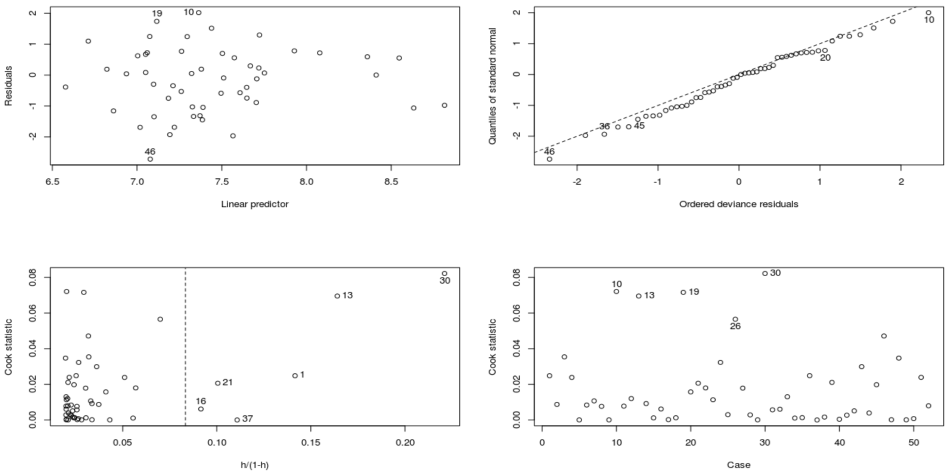

Figure S 4. Diagnostic plots for 4-grams

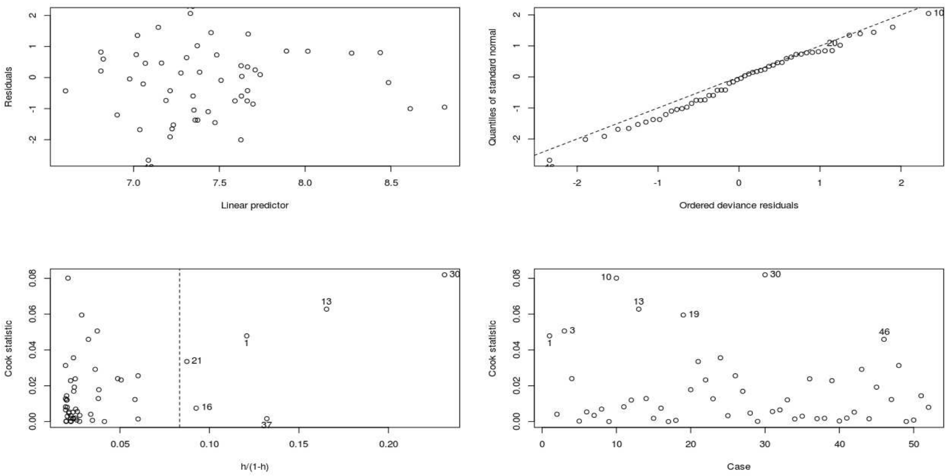

Figure S 5. Diagnostic plots for 5-grams

1.3 Dates of the world's languages

Language group	No of languages	ASJP date	3-grams date	Combined Date
Afro-Asiatic	255	6016	5769	5915
Berber	23	1733	2220	1933
Eastern	3	1697	1159	1476
Northern	15	1158	1750	1401
Tamasheq	4	556	1208	823
Chadic	98	4826	4214	4575
Biu-Mandara	45	4457	3299	3982

Masa	8	1649	1526	1599
West	40	4099	2943	3625
Cushitic	61	4734	3421	4196
Central	8	1686	1493	1607
East	46	3045	3013	3032
South	6	2308	1522	1986
Omotic	31	4968	2622	4006
North	28	3137	2481	2868
South	3	1963	1108	1612
Semitic	40	3301	3234	3274
Central	18	2638	2405	2542
South	22	3804	2557	3293
Khoisan	17	14592	1863	9373
SouthernAfrica	15	5271	1676	3797
Central	7	3143	1223	2356
Northern	3	1846	873	1447
Southern	5	4344	936	2947
Niger-Congo	679	6227	6889	6498
Atlantic-Congo	594	6525	6672	6585
Atlantic	32	2582	2773	2660
Northern	21	6480	2389	4803
Southern	10	5055	1712	3684
Ijoid	34	4546	1831	3433
Volta-Congo	528	5484	6476	5891
Benue-Congo	404	4940	5887	5328
Dogon	10	2202	1471	1902
Kru	5	2317	1012	1782
Kwa	35	4212	2773	3622
Kordofanian	20	4861	2407	3855
Heiban	11	2521	1789	2221
Katla	2	2269	1086	1784
Talodi	6	4658	1507	3366
Mande	64	3417	2520	3049
Eastern	17	1905	1399	1698
Western	47	3047	2257	2723
Nilo-Saharan	149	6642	4563	5790
CentralSudanic	44	5114	2590	4079
East	27	3715	2083	3046
EasternSudanic	68	5988	3667	5036
Eastern	14	5103	2098	3871
Nilotic	47	4508	3152	3952
Western	6	5601	1586	3955
Kadugli-Krongo	11	1221	1641	1393
Komuz	9	5209	1656	3752
Koman	6	2542	1411	2078
Saharan	4	3941	1409	2903
Western	3	3553	1322	2638
Songhai	8	1333	1377	1351
Northern	2	807	859	828

Southern	6	580	1220	842

Table S 2. Dates for language groups of Africa

Language group	No of languages	ASJP date	3-grams date	Combined date
Altaic	79	5954	3236	4840
Mongolic	8	2267	1663	2019
Eastern	7	2145	1562	1906
Tungusic	20	1319	2004	1600
Northern	9	1092	1416	1225
Southern	11	1595	1686	1632
Turkic	51	3404	2430	3005
Andamanese	10	4510	1720	3366
GreatAndamanese	8	2122	1493	1864
SouthAndamanese	2	1186	997	1109
Austro-Asiatic	116	3635	3694	3659
Mon-Khmer	97	3406	3481	3437
Aslian	9	2080	1606	1886
EasternMon-Khmer	41	2479	2372	2435
Nicobar	3	3158	1223	2365
NorthernMon-Khmer	29	3259	2271	2854
Palyu	2	2861	501	1893
Viet-Muong	8	2289	1198	1842
Munda	19	2574	1701	2216
NorthMunda	15	1209	1180	1197
SouthMunda	4	2510	1353	2036
Chukotko-Kamchatkan	5	3368	1781	2717
Northern	3	1192	1471	1306
Dravidian	23	2055	2196	2113
Central	3	695	851	759
Northern	3	2030	994	1605
South-Central	7	2447	1501	2059
Southern	10	1894	1628	1785
Hmong-Mien	14	4243	1420	3086
Hmongic	9	2777	1132	2103
Indo-European	218	4348	4855	4556
Baltic	2			
Eastern	2	1469	1169	1346
Celtic	5			
Insular	5	3876	1547	2921
Germanic	30	1745	2417	2021
North	7	1569	1507	1544
West	23	1398	2110	1690
Indo-Iranian	147	3665	3657	3662
Indo-Aryan	93	1996	3076	2439
Iranian	54	2856	2494	2708
Italic	14			

Romance	14	1759	2136	1914
Slavic	16	1157	2092	1540
East	4	1288	1447	1353
South	6	691	1285	935
West	6	820	1413	1063
Japonic	7	1564	1242	1432
Kartvelian	4	2999	1442	2361
Zan	2	596	1042	779
NorthCaucasian	37	7709	3065	5805
EastCaucasian	32	3907	2863	3479
WestCaucasian	5	3649	1245	2663
Sino-Tibetan	165	5261	4445	4926
Chinese	7	2982	1489	2370
Tibeto-Burman	158	4203	4325	4253
Bai	18	1494	717	1175
Himalayish	54	3182	2944	3084
Karen	10	2345	1148	1854
Kuki-Chin-Naga	18	3411	2122	2883
Lolo-Burmese	9	3436	1471	2630
Nungish	3	1955	675	1430
Tangut-Qiang	3	4660	972	3148
Tai-Kadai	68	3252	2009	2742
Hlai	3	2353	726	1686
Kadai	9	2613	981	1944
Kam-Tai	56	2376	1767	2126
Uralic	24	3178	2666	2968
Finnic	6	876	1278	1041
Mordvin	2	800	1015	888
Permian	3	953	891	928
Sami	6	1532	1564	1545
Samoyed	2	2850	1006	2094
Yeniseian	6	2661	1592	2223
AP	2	2762	1172	2110
KA	2	781	981	863
Yukaghir	2	2027	1162	1672

Table S 3. Dates for language groups of Eurasia

Language group	No of languages	ASJP date	3-gram date	Combined date
Amto-Musan	3	2189	997	1700
Arai-Kwomtari	9	7386	2030	5190
Arai(LeftMay)	4	2974	1358	2311
Kwomtari	5	5968	1686	4212
Australian	192	5296	4534	4984
Bunaban	2	1538	1021	1326
Daly	17	3941	1783	3056
Bringen-Wagaydy	10	2320	1344	1920

Malagmalag	4	1635	1169	1444
Murrinh-Patha	3	2747	1074	2061
Djeragan	2	2750	1240	2131
Giimbiyu	3	415	1130	708
Gunwingguan	25	4517	2714	3778
Burarran	3	3612	1442	2722
Enindhilyagwa	3	4746	1331	3346
Gunwinggic	6	2951	1392	2312
Maran	3	2661	1397	2143
Rembargic	2	1925	1030	1558
Yangmanic	2	1609	1240	1458
Pama-Nyungan	122	4295	3958	4157
Arandic	5	1892	1403	1692
Dyirbalic	4	2137	1369	1822
Galgadungic	2	2366	1063	1832
Karnic	6	2851	1610	2342
Maric	9	929	1290	1077
Paman	21	4918	2403	3887
South-West	23	3103	2453	2837
Waka-Kabic	4	2270	1187	1826
Wiradhuric	3	1129	1193	1155
Worimi	2	2473	1237	1966
Yidinic	2	1237	1015	1146
Yuin	3	1503	1306	1422
Yuulngu	16	1555	1991	1734
WestBarkly	3	2631	1442	2144
Wororan	6	2183	1599	1944
Yiwaidjan	4	2882	1401	2275
Yiwaidjic	2	1407	1066	1267
Austronesian	974	3633	6455	4790
Atayalic	2	2664	1269	2092
EastFormosan	4	2392	1489	2022
Malayo-Polynesian	954	3024	6334	4381
Celebic	61	1796	2565	2111
Eastern	44	1710	2120	1878
Kaili-Pamona	3	1076	1033	1058
Tomini-Tolitoli	12	1468	1705	1565
Central-Eastern	581	3111	5655	4154
CentralMalayo-Polynesian	108	2415	3338	2793
EasternMalayo-Polynesian	472	3803	5426	4468
GreaterBarito	57	2031	2450	2203
East	26	1881	1832	1861
Sama-Bajaw	22	1489	1556	1516
West	8	1087	1428	1227
Javanese	3	566	1030	756
Lampung	24	785	1679	1152
LandDayak	3	1510	1151	1363
Malayo-Sumbawan	34	1845	2445	2091
NorthandEast	32	1898	2365	2089

NorthBorneo	17	2016	2047	2029
Melanau-Kajang	2	1372	946	1197
NorthSarawakan	10	2172	1755	2001
Sabahan	5	1333	1269	1307
NorthwestSumatra-BarrierIslands	4	1822	1193	1564
Philippine	151	1830	3463	2500
Bashiic	10	717	1473	1027
Bilic	8	1633	1397	1536
CentralLuzon	3	1252	1042	1166
GreaterCentralPhilippine	75	1326	2718	1897
Minahasan	5	604	1077	798
NorthernLuzon	42	1621	2337	1915
Sangiric	6	484	1100	737
SouthSulawesi	12	970	1545	1206
Bugis	3	884	1074	962
Makassar	5	558	1140	797
Northern	4	345	1057	637
NorthwestFormosan	2	2204	1220	1801
Tsouic	3	2291	1287	1879
WesternPlains	4	2586	1767	2250
CentralWesternPlains	3	2431	1688	2126
Border	16	3453	2201	2940
Taikat	8	2404	1681	2108
Waris	8	2261	1735	2045
CentralSolomons	5	3677	1403	2745
EastBirdsHead-Sentani	13	6615	2047	4742
EastBirdsHead	3	3590	1299	2651
Sentani	9	4101	1606	3078
EastGeelvinkBay	4	3979	1220	2848
EasternTrans-Fly	39	3257	2359	2889
Kaure	2			
KaureProper	2	2665	1180	2056
LakesPlain	26	5279	2230	4029
Rasawa-Saponi	2	3037	1003	2203
Tariku	22	3541	1999	2909
LeftMay	3	2665	1039	1998
Mairasi	4	1196	1287	1233
Nimboran	5	2059	1220	1715
NorthBougainville	2	2925	1175	2208
Pauwasi	7	4102	1794	3156
Eastern	3	2842	1453	2273
Western	4	1774	1271	1568
Piawi	7	3203	1564	2531
Ramu-LowerSepik	20	6942	2500	5121
LowerSepik	9	3411	2032	2846
Ramu	9	4000	1757	3080
Sepik	28	4827	2693	3952
Ndu	9	1227	1242	1233
Nukuma	2	1791	1105	1510

Ram	2	1791	1006	1469
SepikHill	10	3538	1934	2880
Sko	14	4478	1628	3310
Krisa	8	2400	1315	1955
Vanimo	6	1798	1071	1500
SouthBougainville	3	3054	1273	2324
Buin	2	1744	1135	1494
South-CentralPapuan	20	6232	2326	4631
Morehead-UpperMaro	7	5353	1688	3850
Pahoturi	6	2044	1493	1818
Yelmek-Maklew	4	1468	1074	1306
Tor-Kwerba	14	4435	2106	3480
GreaterKwerba	9	4109	1651	3101
Kwerba	6	3852	1394	2844
Orya-Tor	5	3693	1555	2816
Torricelli	26	5754	2876	4574
Kombio-Arapesh	8	3356	1821	2727
Marienberg	9	3339	1991	2786
Monumbo	2	1867	939	1487
Wapei-Palei	5	5386	1612	3839
Trans-NewGuinea	412	6609	5538	6170
Angan	2			
NuclearAngan	2	4523	1021	3087
Asmat-Kamoro	8	2189	1445	1884
Asmat	4	1033	1074	1050
Sabakor	2	567	891	700
Binanderean	5			
Binandere	5	1842	1366	1647
Bosavi	15	2349	1865	2151
Chimbu-Wahgi	10	3470	1701	2745
Chimbu	5	1635	1266	1484
Hagen	3	1505	926	1268
Jimi	2	912	959	931
Duna-Bogaya	2	3004	968	2169
EastStrickland	7	1401	1297	1358
Eleman	9	4851	1465	3463
NuclearEleman	6	1256	1198	1232
Engan	14	2762	1978	2441
Enga	8	2406	1748	2136
Angal-Kewa	4	1555	1146	1387
Finisterre-Huon	19	4136	2308	3387
Finisterre	5	2868	1428	2278
Huon	14	3044	1995	2614
Gogodala-Suki	8	2827	1440	2258
Gogodala	7	1494	1326	1425
InlandGulf	3	2867	1124	2152
Minanibai	2	2197	981	1698
Kainantu-Goroka	24	4847	2608	3929
Gorokan	14	3186	2248	2801

Kainantu	10	3105	1786	2564
Kayagar	4	1285	1063	1194
Kiwaian	14	1436	1789	1581
Kolopom	3	2892	1113	2163
Madang	101	4573	3852	4277
Croisilles	55	4107	3113	3699
RaiCoast	30	3511	2640	3154
SouthAdelbertRange	15	4165	2197	3358
Marind	14	4014	1848	3126
Boazi	8	1597	1315	1481
Yaqay	3	2069	1086	1666
Mek	4	1309	1294	1303
Eastern	3	1425	1177	1323
Mombum	2	1313	1006	1187
Ok-Awyu	21	4272	2263	3448
Awyu-Dumut	9	2916	1641	2393
Ok	12	2534	1796	2231
SoutheastPapuan	25	5286	2235	4035
Goilalan	2	4233	1119	2956
Koiarian	7	2691	1369	2149
Kwalean	6	3032	1218	2288
Mailuan	3	1238	1042	1158
Manubaran	6	1065	1185	1114
Teberan	2	2322	898	1738
Turama-Kikorian	4	3028	1235	2293
Turama-Omatian	3	1580	1122	1392
West	59	5082	3158	4293
Dani	9	1782	1632	1721
EastTimor	3	1916	1080	1573
WestBomberai	3	3497	1200	2555
WestTimor-Alor-Pantar	40	3531	2665	3176
WisselLakes	3	2060	1091	1663
WestPapuan	33	9083	2408	6346
NorthHalmahera	18	2962	1770	2473
Yele-WestNewBritain	2	6293	1097	4163

Table S 4. Dates for language groups of Pacific

Language group	No of languages	ASJP date	3-gram date	Combined date
Algic	27	5554	3183	4582
Algonquian	25	3343	3059	3227
Central	14	2678	2357	2546
Eastern	8	3026	2216	2694
Plains	2	5002	1151	3423
Caddoan	4	4828	1473	3452
Northern	3	3035	1278	2315
Chumash	5	1792	1426	1642

Eskimo-Aleut	9	5084	1895	3777
Eskimo	8	1842	1816	1831
Gulf	3	7859	1102	5089
Hokan	25	4915	2620	3974
Esselen-Yuman	11			
Yuman	11	1865	1672	1786
Northern	13	5666	2095	4202
Karok-Shasta	5	5246	1748	3812
Pomo	7	1226	1042	1151
Iroquoian	7	4855	1998	3684
NorthernIroquoian	6	3176	1886	2647
FiveNations	5	1673	1672	1673
KiowaTanoan	3	3434	1006	2439
Mayan	76	2220	2738	2432
Cholan-Tzeltalan	9	1432	1386	1413
Cholan	5	1148	1122	1137
Tzeltalan	4	511	1006	714
Huastecan	2	1257	946	1129
Kanjobalan-Chujean	8	1225	1326	1266
Chujean	3	1058	965	1020
Kanjobalan	5	803	1030	896
Quichean-Mamean	52	1649	2135	1848
GreaterMamean	29	1492	1729	1589
GreaterQuichean	23	981	1537	1209
Yucatecan	5	790	1071	905
Mopan-Itza	3	887	959	917
Yucatec-Lacandon	2	601	743	659
Misumalpan	3	2774	1009	2050
Mixe-Zoque	14	1407	1551	1466
Mixe	7	900	1193	1020
Zoque	7	787	1208	960
Muskogean	6	1720	1479	1621
Eastern	4	1188	1285	1228
Western	2	345	981	606
Na-Dene	23			
NuclearNa-Dene	22	8532	2145	5913
Athapaskan-Eyak	21	4203	2073	3330
Athapaskan	20	2062	1956	2019
Oto-Manguean	74	6591	3655	5387
Chiapanec-Mangue	2	2445	1195	1933
Chinantecan	4	1935	1063	1577
Mixtecan	9	4542	1471	3283
Mixtec-Cuicatec	7	3140	1313	2391
Trique	2	1024	801	933
Otopamean	7	3654	1555	2793
Otomian	5	2214	1373	1869
Popolocan	17	3036	1900	2570
Chocho-Popolocan	5	2209	1195	1793
Mazatecan	11	775	1522	1081

Subtiaba-Tlapanecan	6	948	1306	1095
Zapotecan	28	3149	2313	2806
Chatino	3	997	922	966
Zapotec	25	1676	2209	1895
Penutian	25	5522	2833	4420
Maiduan	4	1219	1100	1170
OregonPenutian	4	11886	1510	7632
CoastOregon	3	4902	1399	3466
PlateauPenutian	3	4147	1353	3001
Sahaptin	2	2725	1185	2094
Yok-Utian	11	4413	1943	3400
Utian	9	3663	1805	2901
Miwokan	7	2141	1564	1904
Salishan	20	3827	3041	3505
CentralSalish	10	2459	2131	2325
InteriorSalish	6	2980	1978	2569
Siouan	16	6178	2381	4621
SiouanProper	15	3169	2330	2825
Tequistlatecan	2	1212	997	1124
Totonacan	14	1435	1648	1522
Tepehua	3	506	1237	806
Totonac	11	546	1355	878
Uto-Aztecan	82	4018	3167	3669
NorthernUto-Aztecan	11	2576	1934	2313
Numic	7	1737	1570	1669
SouthernUto-Aztecan	71	3472	2831	3209
Aztecan	58			
GeneralAztec	58	1509	2410	1878
Sonoran	13	2400	1869	2182
Wakashan	5	2781	1377	2205
Northern	2	606	717	652
Southern	3	1154	1225	1183
Yuki	2	2500	1000	1885

Table S 5. Dates for language groups of North and Middle America

Language group	No of languages	ASJP date	3-gram date	Combined date
Arauan	7	1764	1497	1655
Arawakan	49			
Maipuran	49	4134	3460	3858
Aymaran	3	1057	1151	1096
Barbacoan	5	3080	1364	2376
Cayapa-Colorado	2	1419	946	1225
Coconucan	2	419	895	614
Cahuapanan	2	1185	1051	1130
Carib	18	2362	2342	2354
Northern	12	2371	1922	2187

Southern	6	2422	1689	2121
Chapacura-Wanham	2	1931	926	1519
Chibchan	22	4400	2741	3720
Aruak	4	2800	1447	2245
Guaymi	3	3286	1012	2354
Kuna	2	820	1036	909
Rama	2	5117	1124	3480
Talamanca	5	2731	1440	2202
Choco	8	2258	1392	1903
Embera	7	875	1313	1055
Chon	2	2774	1108	2091
Guahiban	5	1291	1537	1392
Jivaroan	4	678	1180	884
Katukinan	3	1965	1074	1600
Macro-Ge	26	7266	2864	5461
Ge-Kaingang	13	4989	1947	3742
Yabuti	2	1607	919	1325
Maku	8	3124	1465	2444
Mascoian	3	1718	1499	1628
Mataco-Guaicuru	10	4701	2110	3639
Guaicuruan	5	2909	1536	2346
Mataco	5	2404	1608	2078
Nambiquaran	3	2807	1235	2162
Panoan	19	1853	2268	2023
North-Central	4	2134	1360	1817
Northern	3	1099	1083	1092
South-Central	6	1853	1532	1721
Southeastern	3	920	1051	974
Quechuan	19	1717	1579	1660
QuechuaII	18	974	1440	1165
Tacanan	4	1590	1203	1431
Araona-Tacana	3	1266	1068	1185
Tucanoan	19	2699	2345	2554
EasternTucanoan	13	1241	1801	1471
WesternTucanoan	5	2156	1597	1927
Tupi	47	3585	3004	3347
Monde	5	1712	1262	1528
Munduruku	2	1480	891	1239
Tupari	3	1850	1033	1515
Tupi-Guarani	32	1550	2492	1936
Yuruna	2	951	836	904
Uru-Chipaya	3	1520	1111	1352
Witotoan	7	5491	1813	3983
Boran	3	2271	1362	1898
Witoto	4	2903	1311	2250
Yanomam	8	1319	1547	1412
Zamucoan	3	2765	1304	2166
Zaparoan	3	3178	1399	2449

Table S 6. Dates for language groups of South America
